# Investigation of *Salmonella* Enteritidis Outbreak Associated with Truffle Oil — District of Columbia, 2015

**DOI:** 10.15585/mmwr.mm6610a4

**Published:** 2017-03-17

**Authors:** S. Janet Kuramoto-Crawford, Sasha McGee, Keith Li, Andrew K. Hennenfent, Kossia Dassie, Jhetari T. Carney, Arian Gibson, Ivory Cooper, Morris Blaylock, Reginald Blackwell, Angela Fields, John Davies-Cole

**Affiliations:** ^1^Epidemic Intelligence Service, CDC; ^2^Center for Policy, Planning, and Evaluation, District of Columbia Department of Health, Washington, DC; ^3^CDC/Council of State and Territorial Epidemiologists, Applied Epidemiology Fellowship, District of Columbia Department of Health, Washington, DC; ^4^Division of State and Local Readiness, Office of Public Health Preparedness and Response, CDC; ^5^Health Regulation and Licensing Administration, District of Columbia Department of Health, Washington, DC; ^6^Public Health Laboratory Division, District of Columbia Department of Forensic Sciences, Washington, DC; ^7^CORE Network, Food and Drug Administration, College Park, Maryland.

On September 8, 2015, the District of Columbia Department of Health (DCDOH) received a call from a person who reported experiencing gastrointestinal illness after eating at a District of Columbia (DC) restaurant with multiple locations throughout the United States (restaurant A). Later the same day, a local emergency department notified DCDOH to report four persons with gastrointestinal illness, all of whom had eaten at restaurant A during August 30–September 5. Two patients had laboratory-confirmed *Salmonella* group D by stool culture. On the evening of September 9, a local newspaper article highlighted a possible outbreak associated with restaurant A. Investigation of the outbreak by DCDOH identified 159 patrons who were residents of 11 states and DC with gastrointestinal illness after eating at restaurant A during July 1–September 10. A case-control study was conducted, which suggested truffle oil–containing food items as a possible source of *Salmonella enterica* serotype Enteritidis infection. Although several violations were noted during the restaurant inspections, the environmental, laboratory, and traceback investigations did not confirm the contamination source. Because of concern about the outbreak, the restaurant’s license was suspended during September 10–15. The collaboration and cooperation of the public, media, health care providers, and local, state, and federal public health officials facilitated recognition of this outbreak involving a pathogen commonly implicated in foodborne illness.

## Epidemiologic Investigation

To identify food items associated with gastrointestinal illness, DCDOH initiated a case-control study; a case was defined as the occurrence of gastrointestinal illness in a person beginning ≤7 days after eating at restaurant A during July 1–September 10, 2015. Cases were categorized as confirmed (*Salmonella* group D isolated from a clinical specimen by culture) or probable (linked epidemiologically, but without laboratory confirmation of *Salmonella*). Case-patients were identified on the basis of laboratory reports confirming *Salmonella*, self-report (i.e., contacted DCDOH directly), notifications from health care providers, and referrals from other restaurant patrons. Control subjects ate at restaurant A during July 1–September 10, 2015, but did not report gastrointestinal illness. Control subjects were identified through case-patients or self-reported to DCDOH. Case-patients and control subjects were interviewed using the DCDOH foodborne investigation questionnaire and were asked to review restaurant A’s online menu and list all food items ordered, shared, or tasted. Sociodemographic and clinical information (e.g., symptoms, doctor visits) was also collected.

During September 9–October 28, 2015, DCDOH identified 277 patrons who ate at restaurant A, among whom 254 (92%) were interviewed directly or through a proxy and included in the analysis. Among the 254 interviewees were 159 (63%) case-patients (40 confirmed and 119 probable) and 95 (37%) control subjects. The majority (90%) of illness onset dates occurred during August 31–September 10 (Figure). Case-patients included DC residents and residents of 11 states, many of whom were visiting DC during the Labor Day weekend. No significant differences were noted between case-patients and control subjects in terms of age, sex, race/ethnicity, and place of residence (Table 1). Among the 153 case-patients for whom symptom information was available, 143 (93%) reported diarrhea, 128 (84%) abdominal cramps, 105 (69%) chills, 103 (67%) headache, 100 (65%) nausea, and 82 (54%) fever.

**FIGURE:**
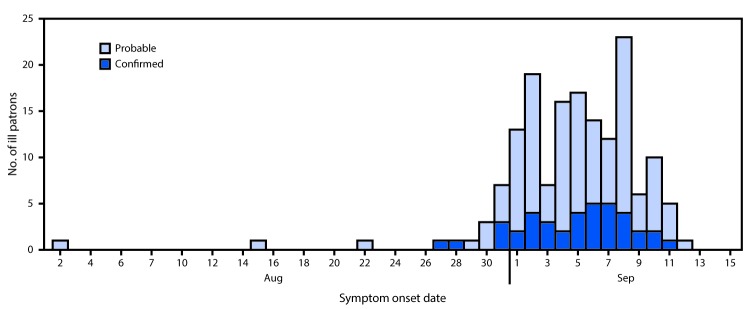
Date of onset* of gastrointestinal illness among 159 case-patients who ate at restaurant A, by case status — Washington, DC, August 2–September 12, 2015^†,§,¶^ * Symptom onset date was missing for six case-patients (five probable, one confirmed). ^†^ One case-patient reported eating at restaurant A twice on the same day and is considered as one entry. ^§^ One case-patient reported two meal occasions at restaurant A (classified as probable on one occasion and as confirmed for another meal occasion) and is counted as two separate entries. ^¶^ Two case-patients reported two meal occasions, but reported illness on only one occasion.

**TABLE 1:** Demographic characteristics of restaurant A patrons (n = 254) during July–September 2015, by case status — Washington, DC, 2015*^,†,^**^§^**

Characteristic	Case-patients (n = 159)^¶^		Control subjects (n = 95)	
	Mean (SD)	Range	Mean (SD)	Range
Age (yrs)	36.6 (11.9)	9–72	38.9 (13.3)	14–80
Female	**No. (%)**		**No. (%)**	
	106 (67)		57 (66)	
White, non-Hispanic	98 (74)		37 (79)	
DC resident**	57 (38)		27 (40)	
Visited doctor	78 (52)		0 (0)	
Hospitalized	9 (12)		0 (0)	
Died	0 (0)		0 (0)	

**Abbreviation**: SD = standard deviation.

* Number of persons with missing information: sex (nine), race (74), age (59), state of residence (36), doctor visits (10), hospitalization (19).

^†^ Seven patrons reported dining at restaurant A on multiple occasions during July–September. Four case-patients reported illness within 7 days after at least one meal occasion, and three control subjects did not report illness on any meal occasion.

^§^ Excludes 21 patrons for whom symptom onset date, meal date, or symptom status were missing and two patrons who were confirmed to be infected with a pathogen other than *Salmonella* group D.

^¶^ Includes 40 confirmed and 119 probable case-patients.

** Case-patients who ate at restaurant A included residents from 11 states and Washington, DC: Washington, DC (57), Virginia (41), Maryland (36), New York (five), Pennsylvania (four), California (two), Alabama (one), Arizona (one), Illinois (one), Kentucky (one), Massachusetts (one), and Michigan (one).

Food items consumed by 155 probable and confirmed case-patients and 88 control subjects were compared. Six food items were significantly associated with case status (Table 2), three of which (beef carpaccio, truffle mushroom croquette, and truffle risotto) contained truffle oil. When all truffle oil–containing items were combined into a single variable, including the three that were individually significant, consumption of a truffle oil–containing item was reported by 89% of case-patients compared with 57% of control subjects (p<0.001).

**TABLE 2:** Selected foods consumed among patrons (n = 243) who reported eating at restaurant A during July–September 2015, by case status — Washington, DC, 2015*^,†,^**^§,¶^**

Food item	Case-patients (n = 155)	Control subjects (n = 88)	p value
	No. (%)	No. (%)	
Burrata crostini	39 (26)	9 (10)	<0.01
Beef carpaccio**	12 (8)	1 (1)	0.04
Branzino	16 (11)	2 (2)	0.02
Lamb chops	14 (9)	1 (1)	0.01
Truffle mushroom croquette**	90 (59)	28 (33)	<0.001
Truffle risotto**	32 (21)	8 (9)	0.02
Any truffle oil–containing item	134 (89)	45 (57)	<0.001

* Four case-patients and three control subjects reported multiple meals during this time period. Two of the four case-patients reported illness on a single occasion and were considered control subjects for the other meal occasion.

**^†^** Excludes four case-patients and seven control subjects for whom information concerning foods consumed at restaurant A was missing.

^§^ Number of patrons with missing information: burrata crostini (14), beef carpaccio (12), branzino (16), lamb chops (16), truffle mushroom croquette (12), truffle risotto (16), and truffle oil–containing item (22).

^¶^ Lists only food items that were significant at p<0.05 by using Pearson’s chi-squared test or Fisher’s exact test.

** Truffle oil–containing item.

DCDOH interviewed six of seven restaurant A employees who reported illness to their manager from late August through early September, the period when most patron illnesses occurred. Two employees sought medical care; one submitted a stool sample for laboratory testing and was confirmed to have a *Salmonella* Enteritidis infection. This employee, who reported eating a truffle oil–containing item that was not offered on the menu in addition to other restaurant A food items, was not involved in food preparation.

## Environmental and Laboratory Investigations

On September 9, a routine restaurant inspection was performed in response to the complaint received the previous day. Although multiple food safety violations were noted, the inspection findings did not warrant restaurant closure. On September 10, a second inspection was conducted as part of the outbreak investigation. Food samples collected on September 9 and 10, and environmental samples collected on September 11 were tested for *Salmonella*. Truffle fries sampled from the deep fryer and uncooked truffle mushroom croquettes were among the samples collected on September 10; a truffle oil sample was collected on September 14. DC Public Health Laboratory (DCPHL) and state public health laboratories performed pulsed-field gel electrophoresis (PFGE) testing on isolates from clinical specimens and uploaded pattern results into PulseNet (*1*). The outbreak cluster code was assigned using clinical samples from two initial hospitalized patients.

DCPHL tested the truffle fries, which screened positive for *Salmonella* by using polymerase chain reaction (PCR), but *Salmonella* was not isolated during confirmatory testing. All other food and environmental samples were negative for *Salmonella*. Among persons who reported illness, 41 (40 patrons and one employee; 26%) had stool samples collected. All 41 had the outbreak *Salmonella* Enteritidis strain (PFGE X*ba*I pattern JEGX01.0008).

## Traceback Investigation

DCDOH issued a nationwide call for cases through CDC’s Epidemic Information Exchange on September 10. Approximately 1 week later, the Los Angeles County Department of Public Health notified DCDOH of a possible outbreak associated with the same restaurant chain at a Los Angeles restaurant. On October 1, the Food and Drug Administration and the New York State Department of Agriculture and Markets inspected the New York-based commissary that prepared and distributed food items to both restaurant locations. Distributed food items to both restaurants were similar and included truffle oil, dried mushrooms, and croquette mix. Food items were unavailable for testing because the commissary had voluntarily ceased operations on September 13. Analysis of 102 subsamples of environmental sponges from food preparation areas using the VIDAS Enzyme Linked Fluorescent Assay did not detect *Salmonella* species. Shipment records for black trumpet mushrooms, cremini mushrooms, truffle oil, and food items prepared at the commissary using these ingredients were reviewed. The records for the implicated truffle oil shipped during August 1–September 15 yielded no significant findings. Truffle oil was regularly shipped to all restaurant A locations across the United States, including locations without any reported illnesses.

## Public Health Response

DCDOH issued a summary suspension of restaurant A’s license on September 10 because of increasing concern about a potential outbreak. Restaurant A removed truffle oil–containing food items from the menu and was required to address food safety risk factor violations before its license was restored. After reopening on September 16, 2015, restaurant A was required to undergo periodic inspections. No additional *Salmonella* Enteritidis cases have been reported since restaurant A reopened.

## Discussion

Gastrointestinal illness was reported in 159 persons from 11 states and DC after eating at restaurant A during July–September, 2015. All confirmed *Salmonella* Enteritidis cases had indistinguishable PFGE patterns. The case-control study results indicated truffle oil as a likely source of infection. Approximately 90% of case-patients reported that they ate a truffle oil–containing item.

Although *Salmonella* Enteritidis is most commonly associated with poultry and eggs (*2*,*3*), the strain identified in this outbreak was also associated with consuming Turkish pine nuts in a 2011 multistate outbreak (*4*). Whole genome sequencing conducted by CDC identified significant differences between this *Salmonella* Enteritidis strain and the one implicated in the 2011 pine nut outbreak. Previous reports indicate that *Salmonella* Enteritidis has the capacity to thrive in low-water activity foods (e.g., nuts and oils) (*5*), including peanut oil (*6*).

The findings in this report are subject to at least three limitations. First, attributing an outbreak to a single food vehicle is a recognized challenge in foodborne outbreak investigations (*2*). In this situation, food and environmental samples were collected after restaurant A had begun disposing of food items and addressing potential sources of contamination, and the commissary inspection occurred after its closure. Second, the truffle oil sampled on September 14 was unlikely to have been consumed by case-patients, because the latest meal date for case-patients was September 9. Finally, because of failure to isolate the organism in culture from food samples, it could not be established whether the PCR-detected *Salmonella* in the truffle fries led to actual illness or matched the outbreak strain. Despite these limitations, the epidemiologic evidence strongly suggested that truffle oil was the likely source of the outbreak.

Recognition of this multistate outbreak associated with truffle oil might have easily gone unnoticed; restaurant patrons and emergency department staff played a significant role in its timely recognition. The PFGE pattern associated with this outbreak is the eighth most common in the PulseNet database. Assigning a specific cluster code for this suspected outbreak at the time isolates from the hospitalized cases were added to PulseNet was difficult because uploads for the pattern code had not exceeded normal thresholds. Close collaboration between DCDOH epidemiologists and DCPHL ultimately led to a cluster code assignment, which facilitated case identification in residents of other states. Results from the routine inspection conducted after the initial complaint did not alone warrant restaurant closure; however, increasing concern about a potential outbreak, based on multiple complaints of illness, prompted DCDOH to suspend the restaurant’s license a day later. This timely public health response likely prevented additional illnesses, because 9% of case-patients reported eating at restaurant A the day before the closure. The engagement of the public, media, health care providers, and local, state, and federal public health officials facilitated recognition of an outbreak involving a *Salmonella* serotype that is a common source of foodborne illness.

**Summary**

**What is already known about this topic?**

*Salmonella enterica* is a common foodborne pathogen, causing an estimated 1 million cases of foodborne illness each year. *Salmonella* Enteriditis is the most common serotype and has frequently been associated with infections attributed to poultry and eggs.

**What is added by this report?**

During July–September 2015, a total of 159 patrons reported gastrointestinal illness after eating at a single District of Columbia restaurant. Forty-one persons (40 restaurant patrons and one employee) were infected with an indistinguishable *Salmonella* Enteritidis strain on the basis of pulsed-field gel electrophoresis (X*ba*I pattern JEGX01.0008). Results from a case-control study using restaurant patron data identified a novel food vehicle, truffle oil, as the likely source of *Salmonella* Enteritidis infection in this outbreak. Approximately 89% of case-patients reported eating truffle oil–containing items, compared with 57% of patrons who did not report gastrointestinal illness (p<0.001).

**What are the implications for public health practice?**

Public health officials and consumers should be aware that truffle oil has been implicated as the likely source of a *Salmonella* Enteritidis outbreak and could possibly harbor this pathogen. Timely engagement of the public, health care providers, and local and federal public health officials, is particularly critical for early recognition of outbreaks involving common foodborne pathogens, such as *Salmonella* Enteritidis.
